# Transposable elements as essential elements in the control of gene expression

**DOI:** 10.1186/s13100-023-00297-3

**Published:** 2023-08-18

**Authors:** Alemu Gebrie

**Affiliations:** Department of Biomedical Sciences, School of Medicine, Debre Markos University, Debre Markos, Ethiopia

**Keywords:** Transposable elements, Mobile elements, Gene expression

## Abstract

Interspersed repetitions called transposable elements (TEs), commonly referred to as mobile elements, make up a significant portion of the genomes of higher animals. TEs contribute in controlling the expression of genes locally and even far away at the transcriptional and post-transcriptional levels, which is one of their significant functional effects on gene function and genome evolution. There are different mechanisms through which TEs control the expression of genes. First, TEs offer cis-regulatory regions in the genome with their inherent regulatory features for their own expression, making them potential factors for controlling the expression of the host genes. Promoter and enhancer elements contain cis-regulatory sites generated from TE, which function as binding sites for a variety of trans-acting factors. Second, a significant portion of miRNAs and long non-coding RNAs (lncRNAs) have been shown to have TEs that encode for regulatory RNAs, revealing the TE origin of these RNAs. Furthermore, it was shown that TE sequences are essential for these RNAs' regulatory actions, which include binding to the target mRNA. By being a member of cis-regulatory and regulatory RNA sequences, TEs therefore play essential regulatory roles. Additionally, it has been suggested that TE-derived regulatory RNAs and cis-regulatory regions both contribute to the evolutionary novelty of gene regulation. Additionally, these regulatory systems arising from TE frequently have tissue-specific functions. The objective of this review is to discuss TE-mediated gene regulation, with a particular emphasis on the processes, contributions of various TE types, differential roles of various tissue types, based mostly on recent studies on humans.

## Introduction

Transposable elements (TEs), also called mobile elements, are DNA fragments that may move about inside a host genome and typically make new copies of themselves while they do so. They are present across all forms of life, accounting for 50% of the mammalian genome [1–3]. TEs are present in the genomes of bacteria, plants and mammals, and are divided in two major classes known as Class I retrotransposons and Class II DNA transposons [4], and these two groups vary from one another in terms of the way they transpose. Class II TEs are less common (3.5%) in the human genome and are regarded as DNA fossils because no family of DNA transposons is still active today [5]. The development of genomics and large-scale functional tests has revealed new knowledge on the many functions of TEs [6].

While DNA transposons move commonly by a cut-and-paste mechanism, retrotransposons do so by a copy-and-paste fashion [7]. The transcription of class I retrotransposons results in an intermediate RNA molecule that may be reverse-transcribed into DNA using reverse transcriptase to create a new copy of the retrotransposon in the genome. On the other hand, Class II DNA transposons produce an enzyme called transposase that separates the parental sequence from the genome before mediating its reintegration into another region of the genome [4, 8].

Retrotransposons come in a variety of forms, such as non-LTR retrotransposons and endogenous retroviruses (ERVs), which are distinguished by the presence of long terminal repeats (LTRs). Long nuclear elements (LINEs), short-interspersed elements (SINEs), and SVAs are further classifications for non-LTR retrotransposons [4, 9]. LINEs make up the majority of non-LTR retrotransposons in the human genome, accounting for 20.4% of it, followed by SINEs (13.1%), LTRs (9.1%), and SVAs (0.1%) [10, 11].

The consequences of TE insertions on host gene expression might be beneficial or harmful, like any mutational process. Regardless of their transposition competency, TE regulatory sequences can be co-opted for host regulatory activities. Evidence gives new thoughts on TE mobility and regulatory potential and serves as a vital resource for population history and disease genetics research [12]. Mechanistically, TEs can influence gene expression either transcriptionally [13], post-transcriptionally [14], or at the step of translation [2, 15] through their encoded products which include both proteins and non-coding RNAs (ncRNAs). More complex than originally thought, the mechanisms by which TEs affect host gene-regulatory networks include: the addition of TFBSs, promoters, and enhancers, alteration of 3D chromatin organization, production of regulatory ncRNAs, co-option/exaptation/domestication of TE-derived coding sequences as new transcriptional effector proteins, and collateral consequences of TE silencing mechanisms [2]. The objective of this review is to discuss TE-mediated gene regulation, with a particular emphasis on the mechanisms, contributions of various TE types, and differential roles of various tissue types, based mostly on recent studies on humans.

## The roles of transposable elements in the human genome and cell

The evolution of genetic information, as well as DNA duplication, stability, and gene expression, are just a few of the numerous facets of DNA function that TEs may affect. The discovery of TEs' involvement in genome evolution and gene function has altered the previously held belief that TEs are junk, parasitic, colonizing, or selfish DNA [16]. New genes with crucial host functions can be produced as a result of TEs [17, 18].

According to a number of studies, TEs play an important role in regulating stem cell characteristics, the epithelial to mesenchymal transition, inflammation, and adaptive characteristics such as elevated gene expression, enhanced gene replication, stress tolerance, and aging [19, 20]. It is shown that the transcriptional control of stress-response genes in *Drosophila melanogaster* is influenced by a variety of families of transposable elements [21]. Also, transposable elements have a significant role in synaptic plasticity, cognition, and tissue development and morphogenesis [22, 23]. In cancer and other inflammatory disorders, the expression of transposon elements triggers a cytokine response and causes the recruitment and infiltration of immune cells [24–28]. In addition to their roles in genomic instability and the trans-regulation of human genes, human endogenous retroviruses have been linked to both the activation and downregulation of the host immune system [29]. A recent study has established how lineage-specific TEs can promote evolutionary turnover and divergence of innate immune regulatory networks and reveals a novel function for B2 SINEs as inducible enhancer elements that affect immunity in mice [30].

Transposons could be altered to incorporate a reporter gene that, when randomly inserted into the bacterial chromosome, can fuse to a gene on the chromosome [31]. This kind of transposon library screening for reporter expression under various situations enables the identification of fusions that are appropriate to stress conditions or a particular therapy. A genome-wide picture of the bacterial regulatory network organization may be obtained from the characterization of these fusions [31]. In addition, a study demonstrated that the expression of retrotransposon is clearly related to aging in Drosophila [32].

## The negative roles of transposable elements in the human genome

Through processes dependent on and independent of transposition, TEs can lead to genomic/epigenomic instability, which may result in different disease conditions, cell death or the development of cancer [20, 26, 33, 34]. The insertion of TEs into the genome's coding regions has the potential to cause missense or non-sense mutations as well as frameshift mutations linked to premature termination. For instance, when *Alu* elements are inserted into mRNA's exonic regions, the open reading frame (ORF) of that specific coding region is altered, which has an impact on gene expression [35]. As Alu elements and LINE-1 can introduce novel splice sites inside an intron, resulting in alternative splicing events that compromise transcriptional integrity, the insertion of TEs into intronic regions can also have negative consequences [35–37]. Additionally, some research has indicated that the insertion of TEs into the 5′ or 3′ regions of genes may impair favorable gene expression [37]. As a result, TE insertions' cumulative effects on gene expression have been linked to a variety of disease conditions, including cancer and genetic disorders [33, 38].

The genomic sequence, chromatin, and nuclear contexts are only a few examples of the factors that interact during TE integration. This variety in insertion-site distribution and evolutionary strategies is explained by these factors [39]. If TEs' capacity to migrate across the genome is not correctly managed, it might be harmful to the host. Long believed to be a mutagen when directed at protein-coding genes, the adverse consequences of mobile element activation were thought to be damaging by triggering chromosomal breakage [40]. TEs typically induce gene disruption and significant genomic abnormalities, including inversions, deletions, and duplications, as a result of their inherent mobility throughout the genome [26].

However, TE transposition may also happen during germline development and, less commonly, in somatic cells [41, 42]. Different instances from various animals and transposon classes have shown the harmful impacts of germline transpositions throughout time. For instance, the P-element DNA transposon (Class II) in Drosophila, which is the source of dysgenic characteristics, and LINE1 (L1; Class I) insertions in human haemophilia A can be taken as examples [43, 44]. Since a growing amount of data could link the somatic transposition of TE with harmful biological consequences, somatic transpositions have also drawn a lot of interest. The mariner-Mos1 element of Drosophila (Class II transposon), which may be transferred during the Drosophila life cycle and adversely influence behavioral activities and embryonic survival [45], is one of the most intriguing examples of this.

There is a plethora of evidence that somatic TE insertions can upregulate oncogenes and lead to genomic rearrangements, which in turn promote different types of cancer [34, 46–48]. Cancer, including ovarian [49], colorectal [50], and Fanconi anemia [51] cancer, to mention a few, is likely one of the clinical conditions that has been most thoroughly studied and associated to a new wave of transpositions in somatic cells. Transposable elements can break free from epigenetic silencing, as has been demonstrated in the majority of cancers. In these cancers, lower methylation levels (hypomethylation) and the dysregulated chromatin modification of L1 retrotransposons lead to their integration into novel sites (insertional mutagenesis), a process that has been observed in pancreatic ductal adenocarcinoma [52] and esophageal squamous cell carcinoma [53].

Through control of cancer-related cellular processes, alternative splicing increases the incidence and progression of several cancer types [54, 55]. The occurrence of alternative splicing events in cancer can be caused by TEs with the genetic capacity to hop to other sections of the genome [56]. By adjusting various mechanisms, including exonization, providing splicing donor/acceptor sites, alternative regulatory sequences or stop codons, driving exon disruption or epigenetic regulation, TEs can integrate into the genome, primarily in the intronic regions, and induce cancer-specific alternative splicing [57]. Additionally, TEs have the ability to create microRNAs (miRNAs), which regulate the number of transcripts by inhibiting translation or promoting transcript destruction at the post-transcriptional level. Notably, TE insertion alters the whole process of gene expression before and after transcription in cancer cells, favoring the growth of the disease [56].

It is now known that TEs are also linked to the development of other brain illnesses, such as autism and schizophrenia [58–61], in addition to cancer. An investigation found 10 polymorphic TE insertions that are plausible candidates for causative roles in neurologic and psychiatric illnesses, on par with other variants [62]. Whole-genome sequencing of schizophrenia patients' brains revealed specific L1 insertions that were preferentially localized to synapse- and schizophrenia-related genes. This raised the possibility that the hyperactivation of L1 retrotransposons plays a role in the susceptibility and pathophysiology of schizophrenia [63, 64]. Transposons have also been suggested to have a part in the pathogenesis of some neurological changes in Fragile X as well as aberrant social behaviors [65].

Additionally, neurodegenerative disorders including Alzheimer's disease have been linked to the pathophysiology of TEs' abnormal activation and mobilization [66]. The differential expression of numerous TEs and the prevalence of neurofibrillary tangles in post-mortem human brains have been linked in an intriguing way by Guo et al. [66], suggesting a connection between TE activation and genomic instability in Tau-mediated AD processes.

Additionally, cortical spreading depression (CSD), an evolutionarily conserved phenomenon that involves a slow, self-propagating depolarization wave that is linked to the spontaneous depression of electrical neuronal activity, has been hypothesized as a neuroprotective mechanism that can silence TEs mobilization through epigenetic mechanisms [67]. Genome destabilization could be resisted by the effects of DNA methylation-mediated epigenetic control of LINE sequence silencing in conjunction with histone modifications in CSD-induced tolerance [68, 69]. This could stop disabling phenomena like senescence.

Furthermore, genes for antibiotic resistance that are transcriptionally silent are activated by transposable elements. The reservoir of transcriptionally inactive genetic material found in bacterial genomes can be triggered by a variety of transposon-related recombination events, which encourage the creation of new drug-resistant bacterial strains [70].

## Mechanisms of transposable elements silencing

**Fig. 1:**
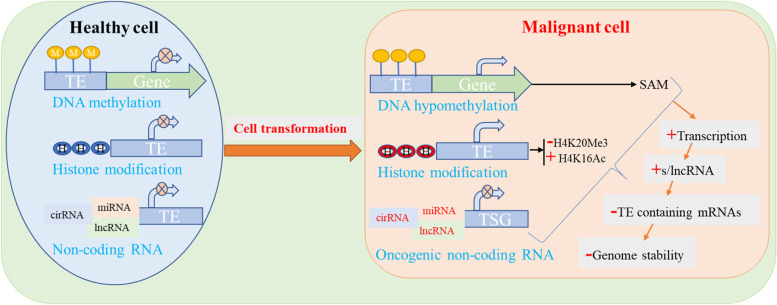
TEs are regulated in both healthy and cancerous cells. Epigenetic changes such as DNA methylation, histone modification, and non-coding RNA (eg cirRNA, miRNA, and lncRNA) inhibit the function of TEs in healthy cells (left panel). During cellular transformation, hypomethylation with increased S-Adenosyl methionine (SAM), various histone modifications (like methylation and acetylation), and oncogenic non-coding RNAs, which inhibit the expression of tumor suppressor genes (TSGs), all contribute to the loss of repressive signals and the uncontrolled production of TEs in cancer cells (right panel). DNA breakdown, mutations, and genomic instability result from all these (arrows indicate the increased activity, cross circle indicates inhibition; ( +) sign indicates increment, (–) sign indicates decrement, and cross sign indicates inhibition) [26]

To prevent the harm brought on by the mobilization of TEs, many protective systems have developed within organisms throughout time [71]. These systems—which are frequently lost in cancer cells—include DNA methylation [72], heterochromatin formation [73], histone alterations [74], and mRNA editing [72]. The majority of TEs in somatic cells are silenced by one of these processes, DNA methylation [75] (Fig. 1).

The interactions between the TEs and a large number of non-coding RNAs are the basis of one well-known germline process [76]. The majority of research on regulatory RNAs has focused on the PIWI-interacting RNAs (piRNAs), which interact with TEs at various levels [77–79]. These RNAs have the ability to silence genes at the transcriptional level or modify the accessibility of proteins to the DNA needed for transcription through epigenetic alterations [80]. They are linked to the RNA-induced silencing complex and have the ability to degrade TE transcripts at the post-transcriptional level by producing double-stranded RNAs (dsRNAs) that can be broken down into small-interfering RNAs (siRNAs). In general, piRNAs function in the gonads to shield the germ-line genomes of both males and females from transposable elements [81]. By altering the histone 3 lysine 9 (H3K9) methylation status, piRNAs suppress TE expression through a mechanism that is consistent across species [82]. Transposon transcription was shown to be enhanced in *Drosophila melanogaster* brains when the Histone 3 lysine 36 methylation mechanism was disrupted [83]. In particular, MIWI2 protein/piRNAs complex recognizes nascent transcripts originating from full-length LINEs and calls for a histone methyltransferase, which deposits the histone 3 trimethyl lysine 9 (H3K9me3) mark on LINE repeats in the germ-line genome [82]. PiRNAs also formed complexes with H1/H3K9me3 and heterochromatin protein 1a (HP1a) in an ovarian somatic cell line, altering chromatin accessibility and influencing TE transcription [84]. Additionally, there is evidence that TEs in *Drosophila* somatic cells are silenced as a result of piRNA [85].

Other molecular processes have also developed, such as TE packing into transcriptionally quiet heterochromatin and TE distribution in areas of low gene density [86]. The targeted accumulation of repressive histone alterations silences TEs. In a variety of cells, tissues, species, and biological situations, DNA methylation has primarily been seen as a technique for preventing transposon movement and maintaining genomic integrity. The fact that TE methylation analyses are frequently used as surrogates for global DNA methylation analyses, given that TEs make up such a sizable portion of the mammalian genome, reflects the close relationship between TEs and their silencing by DNA methylation [87, 88].

In addition, a recent investigation has shown for the first time that Drosophila Fragile X protein is necessary for transposon inactivation in the larval and adult brains of Drosophila "loss of function" dFmr1 mutants [65]. Results from a study also show that the RNA helicase MOV10 inhibits LINE1 retrotransposition in mice in a dosage-dependent way [89].

## Transposable elements in the control of gene expression

If not properly regulated, TE mobilization, expression, and insertion can have detrimental implications on cell physiology. They can also often have a role in regulating gene expression and modifying genomic structure [90]. A ubiquitous mobilization of the L1, Alu, and SINE-R/VNTR/Alu (SVA) transposons, for example, can modify the gene regulatory networks of several types of neurons, notably those in the hippocampus [91, 92]. This is demonstrated in adult brain tissue. Similar to this, several investigations have also shown that L1 retro-transposition in neural precursor cells has a functional purpose [42, 93]. It has been demonstrated, using multi-omic profiling, that L1-promoters are dynamically active in both the developing and adult human brain [94]. Numerous of these transcripts are co-opted as regulatory RNAs or chimera transcripts, and L1s produce hundreds of these developmentally regulated and cell-type-specific transcripts. One human-specific transcript expressed only during brain development is LINC01876, an L1-derived lncRNA. L1s are implicated in human-specific developmental processes as a result of decreased size of cerebral organoids and premature differentiation of neural progenitors caused by CRISPRi-silencing of LINC01876. Therefore, it has been demonstrated that L1-derived transcripts offer a previously unrecognized layer of transcriptome complexity that is unique to humans and primates and contributes to the functional diversity of the human brain [94–96]. Given that TEs can play a dual and contradictory function in the proper differentiation and development of neuronal mosaicism and in the start of neurological illness, the manifestation of TEs in the brain is symbolic of this "double-edged sword" phenomenon.

**Fig. 2:**
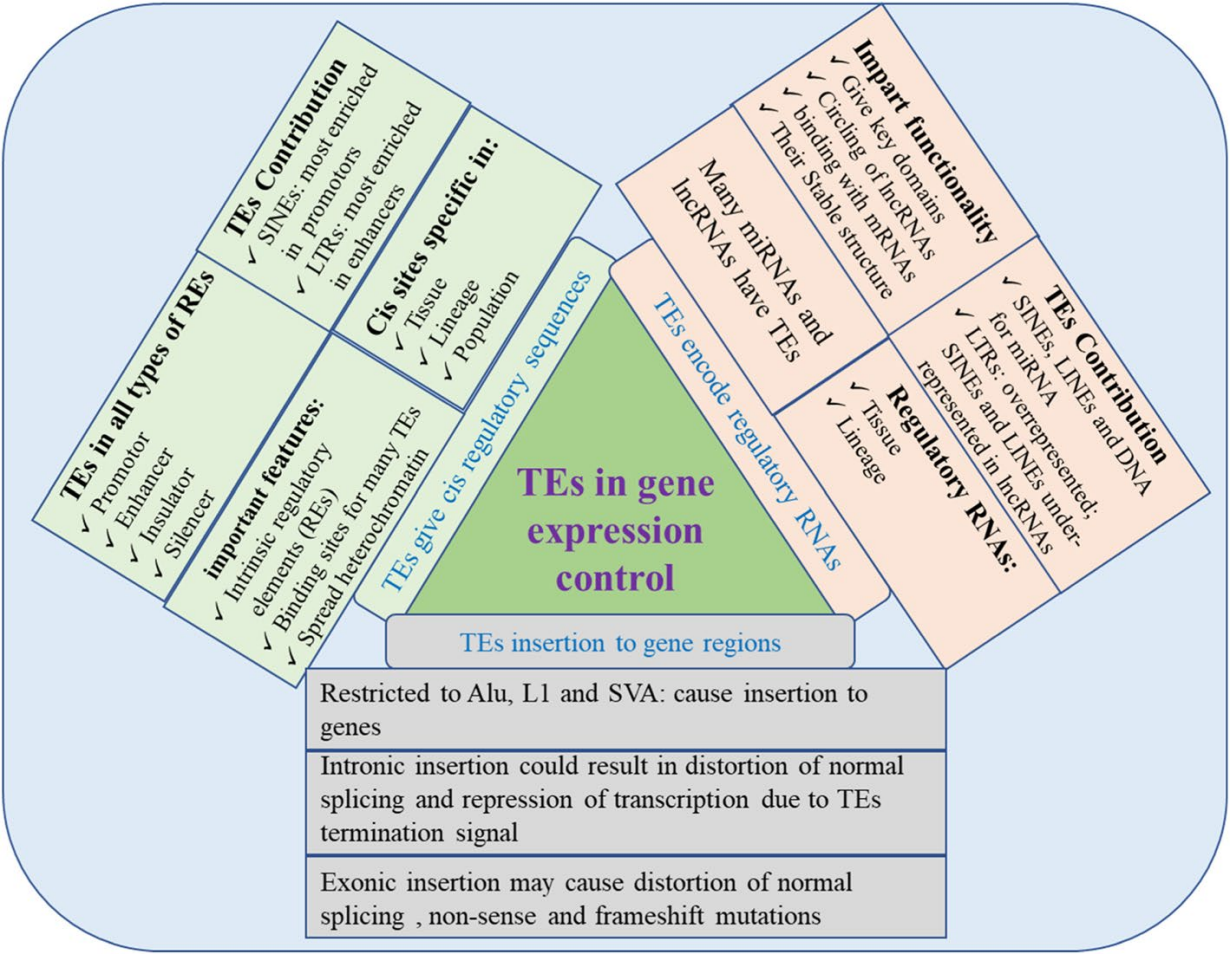
Different mechanisms that TEs influence gene expression regulation

These are intriguing illustrations of how TE evolution has included both mechanisms to prevent these invasive sequences from having a negative impact on genome function and systems to allow them to play an active and beneficial part in it. It is becoming apparent that TEs are a crucial component of the genome's regulatory toolbox [97]. RNA translation, alternative splicing, and gene transcription are just a few biological processes for which repetitive sequences have shown promise as regulators [98]. More and more evidence is mounting that TEs can play a crucial role in regulating gene expression in a variety of mechanisms (Fig. 2).

### The role of TEs in epigenetic gene expression control

Only recently has the role of TEs in 3D genome architecture been studied. TEs have an impact on 3D chromatin architecture with a direct effect on the folding of chromosomes [2, 99]. By serving as insulator elements, TEs can potentially affect the structure of the host chromatin [100–102].

The distribution of TEs has historically been thought to have evolved concurrently with the mechanisms to regulate their expression, and the relationship between TEs and epigenetic modifications has frequently been viewed as consisting of silencing TEs by DNA methylation and histone modification [103]. Transposable elements are also becoming a significant source of epigenetic markers that can affect gene expression, which raises the possibility that TE insertion plays a role in directing epigenetic changes to a particular locus. The cause-and-effect relationship between TE mobilization and distribution and the epigenetic control of gene expression is still a difficult problem. Active intragenic TE insertions preferentially occur into genes in the antisense direction (from 3' to 5') during evolution. This prevents sequence invasion during demethylation waves in the genome, which happen, for example, during the development of germ cells and early embryogenesis [14, 104].

These overlapping sense/antisense transcripts mute the TEs by entering an endo-siRNA pathway that is controlled by DICER and Argonaute 2 (AGO2) and is triggered by global demethylation, which raises the levels of repressive histone marks [104]. It is yet unknown what chemical mechanism causes this correlation between the suppression of TEs and the rise in histone repressive marks [104]. KZFP/KAP1 (Krüppel associated box (KRAB) zinc finger protein/KRAB-associated protein 1) complex plays a crucial role in maintaining heterochromatin, with DNA methylation marks at TEs shielding the loci from TET-mediated demethylation, according to new research in naive murine embryonic stem cells prior to implantation [105]. Ecco et al. [106] discovered that two KRAB/ZFP (Kinc Finger Protein) family members control TE targets by histone-based processes in differentiated tissues, which are not necessarily associated with the DNA methylation state of the loci. Additionally, it has been demonstrated that ZFP92 controls the transcription of particular genes in different tissues by the repression of particular TEs [107].

The work demonstrates that the interactions between the TEs and their KRAB-ZFP controllers affect the expression of neighboring genes. It has been shown that primate-specific ERVs serve as docking sites for the co-repressor protein KAP1 (also known as TRIM28) to produce local heterochromatin in human brain progenitor cells, making this connection even more obvious there [108]. KAP1 binds to the ERVs and represses them, which controls the expression of nearby genes crucial for brain development [108]. The interactions of the transcriptional regulators human silencing hub (HUSH) and microrchidia family CW-type zinc finger 2 (MORC2) with evolutionarily young full-length L1s situated in the transcriptionally permissive euchromatic region, which promotes the deposition of histone H3K9me3, a specific mark for transcriptional silencing, are another example of the regulation of neighboring genes by TEs. A reduction in mRNA expression and potential effects on the RNA polymerase II (POL II) elongation rate might result from this MORC2/HUSH-bound L1 specific impact spreading to nearby genes [109].

Certain kinds of TEs, particularly younger LINEs, have been discovered to affect chromatin accessibility in the livers of several inbred mouse strains, serving as a source of chromatin diversity. This demonstrates the ability of TEs to control tissue-specific genes, which may lead to phenotypic variability among populations [110]. Transposable elements can actively reorganize the chromatin structure to regulate gene expression over a lengthy period of time. About 10% of TE families have been discovered to be enriched in active genomic areas generally and across various organs. While L1 LINEs and ERV LTRs are the most often enriched TE classes in the repressed areas targeted with the H3K9me3 epigenetic mark, SINEs and DNA transposons are the most frequently enriched classes in the active chromatin regions [111].

**Fig. 3:**
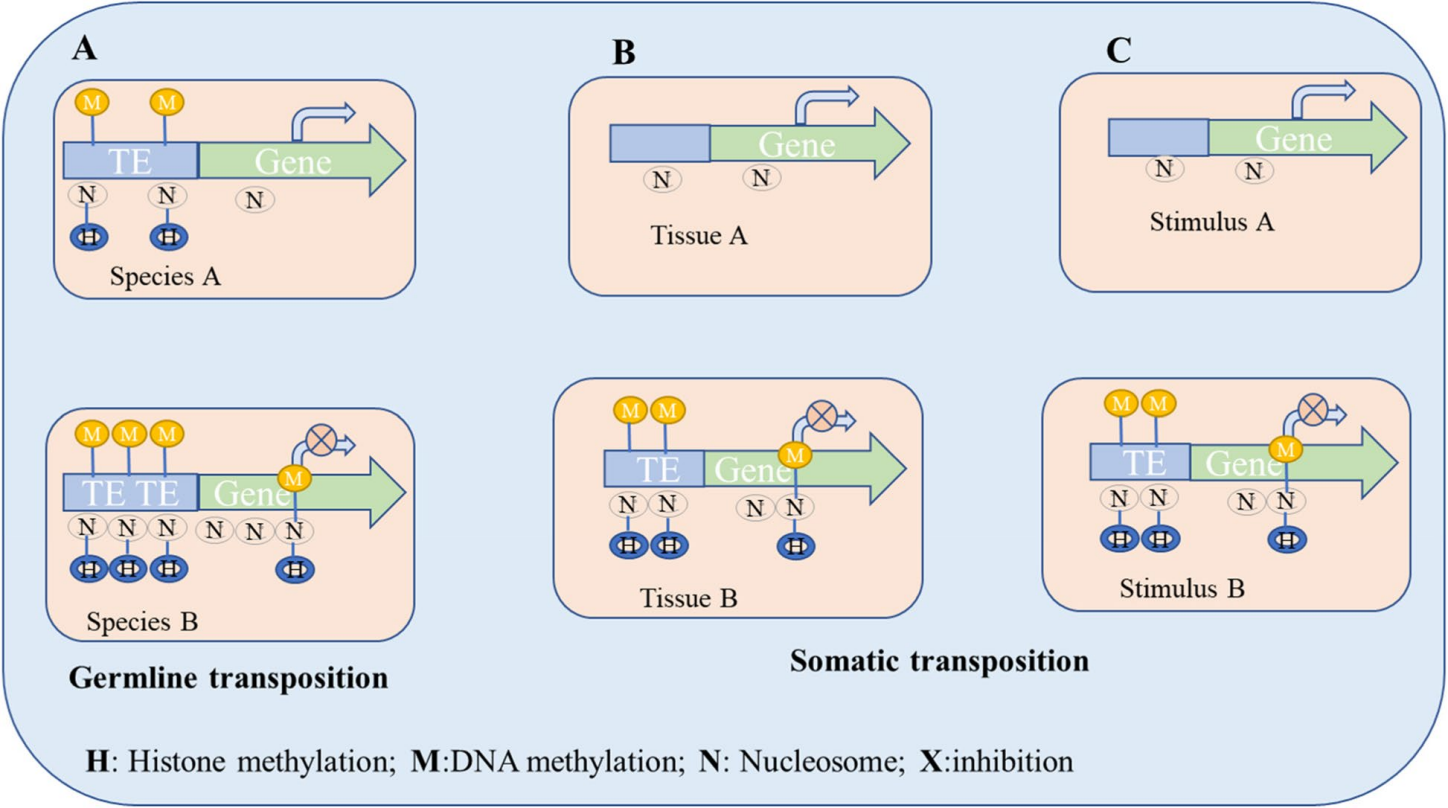
The consequence of TE distribution on the epigenetic control of gene expression due to changes in methylation of histones and DNA across species, tissues of the same organisms, and stimuli or circumstances. **A** The epigenetic control of a particular gene is altered by the varied distribution of TEs in evolution. TE element controls gene expression by influencing the local chromatin state due to changes in methylation of histones and DNA. **B** The expression of a particular gene is impacted by the differential redistribution of TEs in various cells and tissues of the same organism during development. **C** The expression of a certain gene is influenced by the relocalization of TEs sequence in the same cell following a particular stimulus or circumstance

Intriguingly, open euchromatin areas show the strongest epigenetic impact of TEs. By comparing the epigenomes of two *D. melanogaster* strains, it has been shown, for example, that the enrichment of repressive epigenetic marks around euchromatic TEs is caused by the presence of TEs rather than by the preferential insertion of TEs into genomic regions already enriched with repressive epigenetic marks. This pattern explained why TE-flanking alleles had lower transcript levels and greater histone 3 dimethyl lysine 9 (H3K9me2) enrichment than similar alleles without neighboring TE insertions [112]. Similar to this, the analysis of epigenetic marks in flies with and without Bari-Jheh, a natural transposon that affects the expression of nearby genes, revealed significant differences in histone 3 trimethyl lysine 4 (H3K4me3), H3K9me3, and histone 3 trimethyl lysine 27 (H3K27me3) histone marking in relation to oxidative stress conditions, highlighting that this TE element influences gene expression by affecting the local chromatin state. These illustrations imply that the gene expression of neighboring genes is significantly influenced by the various TE distributions seen in the germlines of various organisms/strains and species [113] (Fig. 3A).

The hypothesis that TEs have been co-opted and that their distributions have co-evolved with the control of gene expression is supported by the intriguing fact that varied TE distributions resulting from somatic transpositions impacting gene expression are also relevant in the same individual [114]. In actuality, TE enrichment differs between tissues, and TEs have binding sites for tissue-specific master transcription regulators [111]. The fact that integration is only permitted in open chromatin areas explains one aspect of TE targeting. The vicinity of neuronal genes is where somatic LINE insertions are abundant in mammalian brains.

The non-random and targeted tissue-specific distribution of TEs might be viewed as a way to genetically fix a landmark, which could result in an epigenetic regulation of neighboring gene expression, if we consider that TEs can be the target of epigenetic marks (Fig. 3B). The discovery that various environmental conditions cause L1 transposition through various basic helix-loop-helix PER-ARNT-SIM (bHLH/PAS) proteins raises the prospect of L1 insertions being targeted differently under various forms of stress [115]. Experimental data suggest that TE insertions are targeted in ways that go beyond the ostensible mechanistic need for accessible chromatin [116].

A temporal and functional hierarchy of transcriptional and epigenomic alterations in response to stress is established in Arabidopsis thaliana by the increased DNA methylation that silences TEs near environmental-induced genes [117]. It is feasible to suggest that, in response to particular stimuli, the mobilization and insertion of TEs may also be regulated in adult tissues and post-mitotic cells to drive the epigenetic regulation of particular genes (Fig. 3C).

### Transposable elements in long-range regulation

Different families of TEs have developed many binding sites for transcription factors during the course of evolution, resulting in various transcriptome landscapes [14]. The ENCODE (Encyclopedia of DNA Elements) data comprises roughly 2 million transcription factors binding sites (TFBSs) that coincide with putatively regulation-competent human retrotransposons. For example, these retrotransposons (44% SINEs, 33% LINEs, and 23% LR/ERVs) are situated in a 5-kb gene promoter neighborhood [118]. According to the findings, SINEs are more common than LINE-derived transcription factor binding sites (TFBSs) outside of a 5-kb region close to the transcription start site, but the opposite is true within that region [118].

In addition, while it has long been hypothesized that the repeat sequences that TEs disperse across genomes serve as a source of TFBSs that encourage the emergence of new gene regulatory networks [2], it has only recently become clear that the proteins that TEs encode themselves offer complementary pathways to achieve this result. It was suggested that the process of transposase capture may be a recurring idea in the formation of transcription factors by the discovery that well-characterized transcription factors, such as the paired box (PAX) proteins, feature DNA-binding domains that appear to have arisen from transposases [119].

Additionally, the pathways most significantly influenced by the various retrotransposon distributions have been connected to crucial procedures such as cell stress and immunological responses, ribosome biogenesis, chromatin remodeling, DNA replication, mitotic spindle organization, and cell cycle advancement [118]. The discovery that an evolutionary conserved genomic region called AS3 9, made up of three TEs inserted side by side, serves as a distal enhancer for wnt5a expression during the morphogenesis of the mammalian secondary palate was made by Nishihara et al. [120].

Functional analyses have demonstrated that the AmnSINE1, X6b DNA, and MER117 retrotransposons were co-opted by a retroposition/transposition mechanism during the evolution of mammals. This co-option resulted in the acquisition of a specific Msx1 protein binding site within the X6b DNA sequence, which together with Wnt5a is involved in palatogenesis. According to this study, the great variety of numerous cis-regulatory elements (CREs) may have evolved as a result of the combination of several TEs that were all present in the same DNA segment [120].

### TEs’ role as cis-regulatory elements in the genome

It is believed that the majority of CREs newly evolved during primate evolution are directly derived from TEs [121, 122]. Transposable elements frequently contribute to cis-regulatory elements, tissue-specific expression, and alternative promoters in zebrafish, according to epigenomic analysis [123]. In mammalian genomes, transposable elements are a significant source of various cis-regulatory sequences (Fig. 2). According to some studies, 20% of the CREs found in the human genome may have been taken from TEs [124, 125]. By offering binding sites for trans-acting factors, TEs significantly contribute to all cis-regulatory regions (promoters, enhancers, silencers, and insulators) in the human genome [122]. TEs serve as a reservoir for a variety of regulatory functions and are crucial to the evolution of many regulatory components. They either offer substitute enhancers and promoters or change the activity of the current promoters [126, 127].

It has been well established that TEs may adapt to regulatory elements in the human genome and take on non-TE activities [128, 129]. The transcriptional activity of TE-derived sequences in regulatory elements has been empirically verified in several investigations [127, 130, 131]. According to one study, out of the 35,007 promoters, 75% were identified to have TE-derived sequences, with some promoters possessing as many as ten TEs [132]. However, only 6.8% of the TFBSs in promoters were found to be TE-derived, according to the study.

Studies have shown that TFs bind to TEs and that these proteins contain TF-binding sequence motifs [125, 132, 133]. In the human genome, TFBSs do not just happen to exist across TEs at random. A TF's binding sites are more likely to include copies of particular TE families [133].

Different TE types contribute differently to the regulatory elements in the human genome [134]. While L1s were shown to be least prevalent in the regulatory areas, Alu elements were revealed to contribute the most to all varieties of regulatory regions. Additionally, in gene-surrounding regions, SINE-derived TFBSs outnumber LINE-derived TFBSs, but the inverse is true for regions outside the gene neighborhood [118]. Additionally, contrary to LINEs, SINEs are more common in promoters than in non-promoter areas [132].

It is well known that TE-derived regulatory sequences regulate the expression of a large number of genes in the human genome. By adopting a reporter gene expression strategy or by finding alternative transcripts which start at TE sequences, certain research that concentrated on particular genes were able to uncover TE-derived regulatory elements. For instance, TE-derived regulatory elements control the expression of the Proopiomelanocortin (POMC), Colony Stimulating Factor 1 Receptor, Fc Epsilon Receptor Ig, CD8 genes, and many others [127, 130, 135–137].

Numerous TEs that provide cis-regulatory sequences frequently work in a tissue-specific manner and are crucial for the differential expression of genes in various tissues. Human tissues differ in the epigenetic state of TEs, which affects the profile of TE regulatory functions in various tissue types [138]. One way that TEs are thought to contribute to evolutionary innovation in gene regulation is through their tissue-specificity. Studies that concentrated on certain genes have shown that TEs are adapted to tissue-specific regulatory sequences. For instance, an LTR retroelement offers a neural enhancer for the immunological and POMC genes, and it was shown that Alu sequences give T cell promoters and enhancers for the FCER1 and CD8 genes, respectively [127, 130, 136, 139].

In stem cells, TEs exhibit remarkable cis-regulatory functions. Early germ cells, which share many transcriptional characteristics, rely on a largely overlapping collection of transcription factors, and have widely permissive chromatin landscapes that may further enhance TE activation and have transcriptional activity of TEs that is typically higher [140]. Early in development, young TE families—often LTR elements with embryonic TFBSs in their ancestral sequence—display extremely particular transcriptional patterns [141, 142].

In somatic cells, TEs support cis-regulatory gene networks through the following mechanisms: overlap between the cis-regulatory programs of somatic cells and stem cells, retroviral hijacking of transcription factors expressed in different types of immune cells, or gain of somatic regulatory activity through TE sequence mutations that take place after genomic insertion [139, 143, 144].

Additionally, TE-derived regulatory sites frequently are species/lineage-specific and add innovation and variety to speciation. Future thorough analyses including all regulatory element types across a wide range of species ought to offer more information [127, 145].

However, it has been discovered that mobile element insertion polymorphisms are the most common structural variations in the human genome. Alu elements, L1s, and SVAs are the three groups of retrotransposons that are predominantly in charge of producing human TE polymorphisms [146–149]. The average difference between the two haploid human genomes of the same person is thought to be around 1000 TE insertions [6]. There have not been many studies connecting human polymorphic TEs with variations in gene expression between groups, though.

### mRNA decay and splicing

**Fig. 4:**
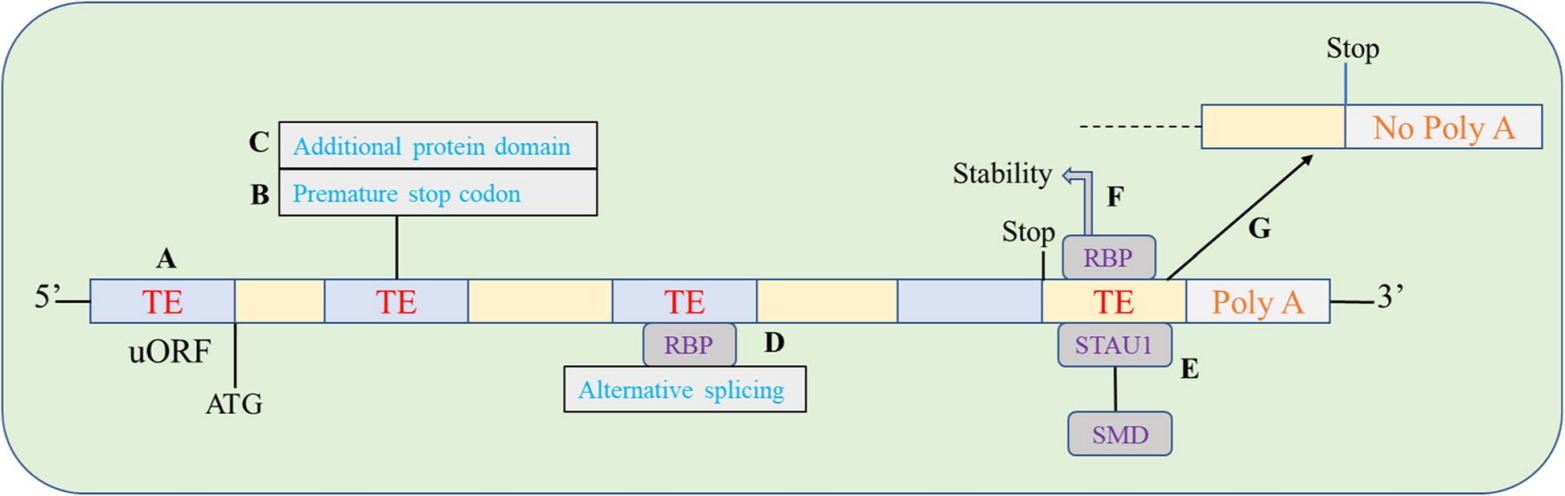
Regulation of gene expression by TE-dependent post-transcription. **A** The presence of an upstream ORF that contributes in the control of the main ORF translation is brought about by a TE inside the 5'-UTR; **B** The existence of an extra domain inside the encoded protein is determined by the exonization of a TE and consequently its translation; **C** The presence of a premature stop codon due to the exonization of a TE inside the coding region of an mRNA can lead to the Nonsense-mediated Decay process; **D** The alternative splicing process can be impacted by TE sequence interactions with RNA-binding proteins (RBP); **E** An mRNA's 3'-UTR contains TE sequences that might cause STAU-mediated degradation; **F** acts as a docking point for RBP important in maintaining RNA stability, such as the HuR protein; **G** or causes the production of a shorter, poly-A tailless mRNA, which results in translational repression

Transposable elements can influence the activity of snc/lncRNAs: microRNAs, circular RNAs, as well as the stability of mRNA through non-sense-mediated decay [14]. Given that a sizable portion of these ncRNAs have their roots in TEs, there is a strong relationship between TEs and regulatory RNAs. The Alu sequences, a subfamily of SINEs elements, are frequently found in introns or the 3′-untranslated region (UTR) of mature and pre-mRNAs [150]. Alu elements included in the sequences of mRNA and lncRNAs may contribute to Staufen-mediated degradation (SMD). Alu elements found in the 3′-UTR of SMD targets and Alu elements found in cytoplasmic and polyadenylated lncRNAs have been shown to imperfectly pair to produce STAU1 binding sites [151]. These locations can interact with the STAU protein, causing a downregulated mRNA expression profile that initiates SMD [151] (Fig. 4).

Lineage-specific 3′-UTR SINEs have a special role in the convergence of gene expression patterns between species, as shown by the ability of these SINEs to control the amounts of mRNAs by directing SMD from orthologous genes in many species (including human) [152]. The control of mRNA quantity and alternative splicing can also be influenced by sequences produced from transposable elements and localized in RNA transcripts. TEs inserted into introns are detected by the splicing machinery and recruited into RNA transcripts as exons in a process known as exonization [14]. TEs have a lot of splice-donor and splice-acceptor sites, which help in alternative splicing. They can thus interact with a wide variety of RNA binding proteins (RBPs) that have particular TE binding sites that they like to attach to, such as the Human antigen R (HuR) or Fused in Sarcoma proteins, which prefer to bind to U-rich motifs [152]. However, the disappearance of TE binding sites for several RBPs suggests that these sites influence transcript quantity and splicing in a manner comparable to that observed in gene-binding sites found in non-repetitive sequences [152]. Additionally, it has been noted that in a small number of instances, the impact of RBP binding might vary according to the particular TE family bound. If it is not bound to an Alu element in a U-rich region, the RBP HuR, for instance, confers transcript stability [153].

Additionally, the Alu-containing RNAs might be able to organize themselves into stable structural domains, which would probably result in new biological activities [154]. By modulating the levels of many nonsense-mediated RNA decay switch exons (NSEs), pseudo-exons produced by the activation of cryptic splice sites that serve as a buffer to prevent Alu-mediated NSE activation, Kralovicova et al. [155] have demonstrated that an intronic transposed element highly similar to medium reiterated frequency repeat family 51 can affect gene expression.

### Transposable elements contribute to noncoding RNAs (ncRNAs)

Noncoding RNAs, which are often longer than 200 nucleotides and are not translated into proteins, include long non-coding RNAs and small non-coding RNAs [156, 157]. There are several types of long non-coding RNAs, each with a unique genomic location in regard to genes and exons [158]. These include Long intervening/intergenic noncoding RNAs which do not overlap protein-coding genes, intronic ncRNAs, and sense and antisense lncRNAs. On the other hand, small ncRNAs consist of a variety of RNAs, including piwiRNAs, microRNAs, and small nucleolar RNAs [157, 159].

There is no doubt that TEs have significantly influenced regulatory RNAs (miRNAs and lncRNAs) [160, 161] (Fig. 2). Certain TE families' palindromic sequences play critical roles in the hairpin structure of miRNAs, and various TEs are connected to various miRNA families. An investigation on the origins of human miRNAs from transposons revealed that miRNAs are most frequently produced from LINE elements (108 miRNAs) and SINE elements (94 miRNAs), and less frequently from DNA transposons (64 miRNAs) and LTR-containing retroelements (53 miRNAs) [162]. Additionally, mature miRNAs that are not hairpins contain TE sequences. The fact that TEs are found in promoters, introns, and exons of lncRNA genes emphasizes the role that TEs play in the production of lncRNAs [163, 164].

Compared to protein-coding genes, long non-coding RNAs (lncRNAs) have a substantially greater density of TE-derived sequences, with TEs making up around 30% of the total lncRNA sequence in human and mouse [165, 166]. TEs offer significant cues for the synthesis of lncRNAs, just as they do for protein-coding genes. For instance, LTR-derived promoters control the expression of 10% of human lncRNAs [165]. Additionally, many ways by which TE sequences produced as a component of lncRNA species might alter gene expression [167, 168]. Numerous lncRNAs produced from TE have been linked to embryonic development. The transcriptome of pluripotent stem cells has been found to be 30% more complicated than that of differentiated cells, likely as a result of the prominent transcriptional activation of TEs [169]. Of note, some are significantly expressed in these cells. The variety of molecular methods that may be used to analyze TE-derived ncRNAs has skyrocketed in recent years. As a result, it can be anticipated that there will be plenty of intriguing and novel roles for ncRNAs originating from TEs still to be uncovered.

The circular RNAs (circRNA), a novel family of short noncoding RNAs with gene control activities, are another class of small RNAs implicated in TE regulation. Recent mammalian investigations have demonstrated that transposons with the capacity to drive circRNA synthesis via reverse complementary pairing are abundant in the flanking regions of circRNAs [170, 171]. A variety of Alu pairings are found in human introns when multiple circRNAs derived from the same gene locus are present, pointing to a potential role for pairing competition in the development of alternative circularizations [172]. LINE1-like elements and their reverse complementary pairs (LLERCPs) are highly abundant in the flanking regions of circRNAs, according to recent research in maize that sequenced circRNA [173]. It is interesting to note that when LLERCP transcription rises, circRNA accumulation changes and linear transcript levels fall [173].

The expression of a different class of long non-coding RNAs known as cis-natural antisense transcripts (cis-NATs) can be influenced by TEs in various ways, according to Jung et al. [174]. First, depending on the TE, different promoters can be used to transcribe NATs that come from TEs. They can also be exonized by TEs, with the newly generated exon complementing the exon of a gene encoding a sense protein [174]. Thus, NATs may use dsRNA formations to engage in RNA interference or adenosine deaminase acting on RNA (ADAR) pathways to mediate the production of sense transcripts.

The results of several studies demonstrate that TEs have a role in how regulatory RNAs work, including, but not limited to, assisting circularize lncRNAs, binding regulatory RNA to target mRNAs, and creating stable secondary structures for regulatory RNAs [175, 176]. Different sncRNA and lncRNA types receive functional features from TE-derived sequences, which makes them crucial for regulatory RNA activities [170, 177].

The lineage and tissue specificity of TE-derived regulatory RNAs has been shown in several investigations. Because of their tendency to be less conserved and more lineage-specific, TEs are thought to be a significant source of the lineage-specificity of regulatory RNAs [163, 167, 178–180]. Also, TE-enriched regulatory RNAs can be tissue-specific, according to research. For instance, in the work by Kang et al., it was discovered that a total of 29 human lncRNAs had tissue-specific expression, of which 20 were lncRNAs originating from TE [174]. Additionally, TE sequences are present in 9 out of the 11 lncRNAs that have been shown to be expressed in cancer cell lines, indicating that these lncRNAs play a role in the development of cancer [166, 181, 182].

SINEs and LINEs make up the majority of TEs that contribute to the sequence of lncRNA. SINEs and LINEs are underrepresented in lncRNAs compared to the whole genome, while LTRs are overrepresented. In conclusion, the distribution of TEs in the introns of lncRNA genes is essentially similar to that of the entire genome, but LINEs are underrepresented in exonic and promoter areas while LTRs are overrepresented in lncRNA exons and promoters compared to protein-coding genes [166, 179, 183].

### Transposable elements and protein translation

Retrotransposons have developed alongside genes, inserting into various locations along the gene bodies and resulting in a wide range of outcomes (Fig. 2). The expression of many genes' proteins is affected by transposable element insertions into mRNAs' 3′-UTRs or 5′-UTRs in a variety of ways. According to Kitano et al.’s research [184], TEs are also involved in the translational control of several genes through the use of upstream open reading frames (uORFs). One study's findings point to a transposon as the source of uORFs and reveal a novel function for transposable elements in influencing protein abundance and phenotypic variety by altering translation rate [185]. Canonical ORFs located downstream of the uORFs in eukaryotic mRNAs can act as cis-acting elements to inhibit or promote translation through the use of the major families of retrotransposons, such as LINEs and SINEs.

Kitano et al. [184] used the human RefSeq mRNA sequence database to show that 10% of human uORFs are produced and controlled by TEs located in the 5′-UTR of mRNAs. Although earlier research has shown that retrotransposons function as translational regulators, it is still unclear how DNA transposons affect the translation of the protein-host. The genomes of both plants and animals include a large number of MITEs, or miniature inverted-repeat transposable elements. Their presence in the 3′-UTRs of rice mRNAs has been shown to have a regulatory effect via a translational repression mechanism. In rice, the Ghd2 gene, a member of the CCT gene family (CONSTANS (CO), CO-Such, and TIMING OF CAB1), controls crucial agronomic variables like grain quantity, plant height, and heading date [186]. The Dicer-like 3a (OsDCL3a) pathway, which may produce shorter mRNA without a poly-A tail by processing the nascent MITE transcripts, is the method by which the MITEs inhibit Ghd2's translation [186]. It is still unknown how MITEs suppress mRNA translation and which phase of mRNA translation is impeded by them [186]. TEs have been linked to the creation of novel alternative mRNA splicing isoforms when found in the coding region of genes.

This can be viewed as a transitional stage in the evolution of new genes. Examples include the lamina-associated polypeptide 2alpha (LAP2alpha) domain-containing splice isoforms of the mammalian thymopoietin (TMPO) and zinc finger protein 451 (ZNF451) genes, which are both related to the first ORF from a retrotransposon-like Dictyostelium intermediate repeat sequence 1. The canonical protein and a brand-new non-canonical protein isoform are both produced by both mRNAs. Particularly, during evolution, the LAP2a specific isoform of TMPO was co-opted to support a new and significant role in the cell [54, 187].

In a process known as domestication, the evolutionary insertion of TEs in gene coding areas has also resulted in chimeras. An excellent example of retrotransposon domestication is the activity regulated cytoskeleton associated Protein (Arc), which most research indicates is descended from a vertebrate lineage of Ty3/gypsy retrotransposons [187]. Particularly important for learning and memory is the cellular immediate-early gene Arc, whose mRNA is found near the synaptic junction. Arc possesses an internal ribosomal entry site that permits cap-independent translation, which is an intriguing way in which the control of Arc mRNA mirrors that of viral RNA [188].

The protein structure of the Arc subdomains has been demonstrated by crystallography to create a bi-lobar architecture akin to the capsid domain of the human immunodeficiency virus gag protein [188]. These results imply that Gag-containing components have been repurposed by evolution to mediate intercellular communication in the neurological system [188].

## Therapeutic potential of TEs

Transposable elements might serve as therapeutic targets for a variety of complicated diseases, including malignancies and CNS-related genetic disorders. Transposable elements are capable of sequence insertion or deletion, enabling precise control of gene expression and modifications to pathophysiological pathways. The creation of specialized oligonucleotide sequences aimed at these sequences may be made possible by the identification of certain TEs involved in the etiology of the diseases. The potential of TEs as gene therapy tools has been examined in a number of preclinical investigations using various models of human disorders [26]. It is noteworthy that gene therapy employing TE-based vectors has shown to be a promising approach for the treatment of many hereditary and acquired human disorders. In addition, many DNA-transposon-based vectors were modified for gene therapy procedures by taking use of qualities like its potential for integration and non-viral nature [189].

Current clinical studies frequently target TEs or benefit from TE biology. Clinical studies using checkpoint inhibitor treatment for immune signaling against renal, ovarian, colorectal, and melanoma malignancies that include TE signaling pathways are currently being conducted [190].

In relation to TEs, both humoral and cell-mediated immunity have been investigated. Several malignancies, including ovarian and melanoma patients as well as teratocarcinoma cell lines, have been linked to anti-ERV-K antibodies [191]. Adaptive immune activity to target TEs as new therapeutic targets was found to be aided in cancer patients by T-cell-mediated and autologous humoral response.

Overexpression of transposon elements in different human diseases is due to demethylation of the TE loci [192]. The TE transcript mechanism, however, is occasionally independent of DNA methylation [193]. This raises the possibility of additional TE regulatory mechanism for non-coding RNAs and histone alterations. Human disorders are significantly influenced by RNA modification [194]. It has been revealed that transposon RNA M(6)A underwent one of its modifications [195]. Transposons may have a role in certain human disease mechanisms, by making use of attractive targets for treatments. In addition to RNA changes, one may look at the uncharacterized DNA modifications of TEs for additional study. One such is m6dA, which is found in the human genome at certain locations, is linked to enhanced transcription activity, and has been implicated in cancer [196, 197]. The link between TE loci and biomarkers raised in disease states would be an intriguing area for further research.

## Limitation of the review

This review has a limitation in that it non-specifically addresses the role of transposable elements in the regulation of gene expression. It is more descriptive since it is a narrative review rather than a systematic review and/or meta-analysis, which are supported by statistical analyses and which can objectively answer a particular subject. Therefore, this review presents the authors' own perspectives on a more general topic.

## Conclusion and perspective

There are a number of recent discoveries that support the increasingly clear active involvement of TEs in genome function, highlighting their impact on the control of gene expression. In addition to providing ready-to-use TFBSs or undergoing mutations to generate binding motifs for TFs, TEs have inherent regulatory mechanisms for controlling their own expression. Many genes' regulatory elements contain TE sequences, which are involved in both short- and long-range regulation of gene expression. By actively taking part in the production of regulatory RNAs, TEs also contribute to the control of genes. There is still much to learn about the function of transposable elements in gene regulation and their therapeutic potential.

## Data Availability

Not Applicable.

## Abbreviations

bHLH: Basic helix-loop-helix
CD8: Cluster of differentiation 8
CircRNA: Circular RNAs
cis-NATs: Cis-natural antisense transcripts
CREs: Cis-regulatory elements
CSD: Cortical spreading depression
H3K9: Histone 3 lysine 9
HP1a: Heterochromatin protein 1a
HuR: Human antigen R
HUSH: Regulators human silencing hub
KRAB: Krüppel associated box
LINEs: Long interspersed nuclear elements
LLERCPs: LINE1-like elements and their reverse complementary pairs
LTRs: Long terminal repeats
miRNAs: MicroRNAs
NSEs: Nonsense-mediated RNA decay switch exons
piRNAs: P-element Induced WImpy testis (PIWI)-interacting RNAs
POMC: Proopiomelanocortin
RBPs: RNA binding proteins
s/lncRNAs: Short/Long non-coding Ribonucleic acids
SINEs: Short-interspersed nuclear elements
SiRNAs: Small-interfering RNAs
SMD: Staufen-mediated degradation
SVAs: SINEs Variable Number of Tandem Repeats Alu
TEs: Transposable elements
TFBSs: Transcription factor binding sites
UORFs: Upstream open reading frames
UTR: Untranslated region
